# Aging is not a disease: an evolutionary and comparative biological reappraisal

**DOI:** 10.1007/s10522-026-10422-3

**Published:** 2026-03-23

**Authors:** Bruno César Feltes

**Affiliations:** Institute of Biosciences, Department of Biophysics, Universidade Federal do Rio Grande Do Sul – UFRGS, Avenida Bento Gonçalves 9500 - Prédio 43422, Sala 218, Porto Alegre, Rio Grande do Sul 91509-900 Brazil

**Keywords:** DNA repair, Evolution, Aging, Disease classification, Inflammaging, Disease burden, Evolutionary theory of aging, Disease

## Abstract

The question of whether aging should be classified as a disease has gained prominence in geroscience, fueled by advances in molecular biology and the aspiration to develop interventions that mitigate age-associated functional decline. However, evolutionary models describe aging as an emergent consequence of declining selection gradients and life-history trade-offs rather than as a deviation from species-typical function. Comparative data across taxa reveal substantial heterogeneity in aging trajectories, challenging the assumption of a uniform pathological pattern. At the molecular level, processes often described as “hallmarks of aging” reflect conserved regulatory mechanisms whose effects are context-dependent and do not consistently align with criteria used to define diseases. Likewise, epigenetic clocks capture molecular signatures that track biological aging and predict mortality risk, yet these biomarkers reflect time-dependent molecular remodeling rather than an underlying pathological process. While translational research aimed at extending healthspan has generated important advances, current empirical evidence does not support equating aging with disease in a strict biological sense. Even though the classification of aging is not relevant to the importance of investing in aging research to alleviate its burden, maintaining a clear conceptual distinction between time-dependent biological remodeling and pathological dysfunction may provide a more coherent basis for both scientific inquiry and therapeutic development.

## Introduction

Aging is the most universal and significant risk factor for numerous diseases in humans, including cancer (Trastus and d’Adda di Fagagna 2025), dementia (Feigin et al. 2019; Nichols et al. 2022), diabetes (Bellary et al. 2021), autoimmune diseases (Liu et al. 2023), and metabolic disorders (Amorim et al. 2022), among others. In other words, even if individuals lead healthy lives, as they age, their likelihood of developing diseases and infections increases, and their overall biological fitness declines.

This started debates about the definition of the aging process. Discussions on this topic feature a variety of perspectives and continue to grow as more empirical evidence contributes to the overall understanding (Rose et al. 2012; Lemoine 2020; Menassa et al. 2023). Nonetheless, most researchers generally agree that the aging process encompasses a series of complex, time-dependent changes occurring at multiple levels within an organism. These changes are typically viewed as damaging, leading to a gradual deterioration in the functionality and effectiveness of biochemical processes. As these processes deteriorate, the organism's ability to maintain homeostasis and respond to environmental challenges diminishes, contributing to a decline in overall health and vitality.

Because of its dynamic and multi-level nature, aging cannot be explained from a single perspective, leading to numerous theories to understand aging (da Costa et al. 2016). These theories seek to elucidate the mechanisms underlying physiological deterioration throughout life and to identify the factors that accelerate this process. While major theories of aging are only one aspect of a complex process, several key molecules and pathways repeatedly emerge as essential factors in the decline of cellular functions across organisms (López-Otín et al. 2013, 2023). Due to the consistent dysregulation of certain molecular pathways during the aging process across species, questions have emerged: Is aging programmed to occur? If not, can it be halted or reversed?

Given the complex dynamics, shared hallmarks, outcomes, and molecular changes of the aging process across organisms, the investigation of its origins has become a noteworthy focus of gerontology (Li et al. 2023; Pamplona et al. 2023), second only to our need to treat aging-associated diseases. Among the debated origins of aging, a discussion emerged that aging aligns with disease criteria, following five major converging arguments. First, is that aging shares molecular and cellular hallmarks with recognized pathologies, including genomic instability, chronic inflammation, and cellular senescence. Second, as the leading risk factor for most chronic conditions, aging is viewed as a primary target for prevention. Third, pharmacological interventions that modulate aging-associated biomarkers are considered evidence of therapeutic efficacy. Fourth, formal disease classification could facilitate regulatory approval and funding. Finally, it is argued that the progressive decline in physiological function may constitute a deviation from optimal biological performance, leading to suffering and impairment.

The answer divides the biomedical community into those who view aging as an irreversible, but manageable and delayable process, and those who seek to classify it as a treatable disease. This review engages these arguments. Rather than dismissing them, I evaluate whether they reflect the criteria required for disease classification. Examining the classification question does not imply opposition to interventions to extend healthspan, because it is clear that we must pursue ways to alleviate the physiological burden of aging. However, conceptual precision may strengthen translational efforts by clarifying targets and biological expectations.

## Evolutionary data impacts the classification of aging as a disease

The debate over what qualifies a condition as a disease has been ongoing for decades. While some authors have proposed that senescence can be understood as a syndrome of age-associated pathologies whose incidence increases with chronological age (Gems 2015), this does not logically imply that aging constitutes a disease. Conversely, proposals to classify aging as a disease within the context of ICD-11 frequently rely on institutional and economic arguments rather than biological criteria (Zhavoronkov and Bhullar 2015). Hence, the existence of pathological processes during aging does not, by itself, justify redefining aging as a disease, particularly when the boundaries, biomarkers, and onset of such a condition remain undefined. Likewise, the prospect of potential economic benefits does not override the need for conceptual clarity grounded in evolutionary and biological data.

In this sense, modern definitions in evolutionary medicine, as broadly defined and discussed by Boorse, converge on the view that a pathological condition represent “*a state of statistically species-subnormal biological part-function, relative to sex and age*”, where “subnormal” means “*far below the mean of function in a typical environment*” and are, in principle, subject to negative selection (Boorse 1977, 2014). Boorse broadly discusses that there are specific physiological variations in physiological metrics (heart rate, temperature, etc.) within a species, depending on lifestyle (e.g., athletes, people living at different altitudes, night workers), but such variations do not constitute the whole class of the species, indicating that the negative impact of any physiological variation must be overall universal to be subnormal (Boorse 2014). Likewise, some variations in physiological parameters do not directly imply impairment, suffering, or physiological decline, as aging is experienced differently by each individual. For a complete assessment of the debate, refer to (Boorse 2014, 2025).

Based on ICD-11 and numerous age-related releases, the World Health Organization (WHO) makes a clear distinction between disease and aging. In this context, diseases are understood as conditions involving clinically relevant dysfunction of biological or psychological processes, associated with impairment, suffering, or increased risk of mortality, and amenable to clinical diagnosis and intervention. However, WHO did not add aging as a disease in ICD-11 and clearly states that biological aging is a normal part of life, not something to be diagnosed (World Health Organization 2015; The World Health Organization 2019). Importantly, WHO has explicitly avoided classifying aging itself as a disease and has explained that aging is experienced differently by each individual, without a defined progression and outcome, as is expected of a disease (The World Health Organization 2020, 2025, 2026). During the development of ICD-11, a proposal to include “old age” as a diagnostic category was withdrawn and replaced by “decline in intrinsic capacity,” which captures age-related functional changes without labeling them as pathological (The World Health Organization 2026).

The progressive decline in physiological homeostasis that we designate as aging, whether expressed through recognizable phenotypes or through molecular remodeling, is observed across virtually all animal taxa. Despite efforts to label aging as a disease, evolutionary theory consistently treats it as a byproduct of natural selection shaped by environmental contexts, making such a classification incompatible, even though aging strongly modulates disease risk. It should also be recognized that human aging occurs within complex social and environmental contexts that extend beyond the biological processes typically studied in experimental organisms. Socioeconomic conditions, psychological stress, cultural factors, and technological environments can influence health trajectories (Epel and Prather 2018; Araya-Ajoy et al. 2020; Kivimäki et al. 2025). These dimensions are determinants of human aging but are distinct from the biological mechanisms underlying organismal senescence. For this reason, the present discussion focuses primarily on the evolutionary and physiological aspects of aging biology, while acknowledging that human aging ultimately emerges from the interaction between biological processes and broader environmental and societal influences.

Medawar first proposed that the declining force of natural selection with age allows late-acting deleterious mutations to accumulate in populations (Medawar 1953). Williams extended this reasoning through the antagonistic pleiotropy hypothesis (APH), suggesting that alleles conferring early-life reproductive advantages may be favored even if they produce harmful effects later in life (Williams 1957). Kirkwood further formalized these ideas in the disposable soma theory (DST), arguing that organisms allocate limited energetic resources between reproduction and somatic maintenance, leading to gradual deterioration as an optimized evolutionary trade-off (Kirkwood 1977). These theories formed the backbone of the hundreds of theories that emerged in the ever-expanding universe of aging research and grounded aging as a selective trade-off, a theme echoed by all other theories (da Costa et al. 2016). A key dynamic, however, is recognizing that historical environmental context, whether social or ecological, plays a pivotal role in shaping an organism's functional decline. In this context, aging is highly heterogeneous and does not appear to be a universal downhill process of deterioration across all organisms.

Jones et al. examined age-specific mortality and fertility trajectories across 46 species, including mammals, birds, reptiles, amphibians, invertebrates, plants, and even algae (Jones et al. 2014). Contrary to the long-standing assumption that senescence universally involves progressively increased mortality after reproductive maturity, the authors found heterogeneity in aging patterns. In several species, mortality rates remain constant with age; in others, they decline, a phenomenon described as negative senescence. Fertility trajectories are equally diverse, with some species maintaining or even increasing reproductive output in later life stages (Jones et al. 2014). While some opinions use such variation of mortality as proof that aging can be reversed or halted, these interspecies patterns align with evolutionary predictions that aging reflects species-specific optimization strategies within given environmental contexts. Importantly, the authors explicitly caution against anthropocentric interpretations, emphasizing that modern humans are demographic outliers with unusually steep increases in late-life mortality. Hence, if aging were analogous to a disease, we would expect consistent deterioration across taxa and a defined onset.

In this sense, demographic analyses of supercentenarians show that, after approximately age 110, the annual probability of death plateaus at around 50%, rather than continuing to rise exponentially as predicted by classical mortality models (Vaupel 2010). The sparse survival data beyond age 114 are consistent with the hypothesis that mortality virtually remains at this level. This suggests that individuals who survive to extreme ages arrive in a more robust physiological state. At advanced ages, remaining individuals appear to deteriorate at broadly similar rates.

Moreover, a study used multivariate biomarker data from large human cohorts and demonstrated that physiological dysregulation can be better understood as deviations in integrated biomarker networks rather than as abnormalities in single molecular variables (Cohen 2016). One key axis, consistent across cohorts, reflects coordinated variation in albumin, hemoglobin, inflammatory markers, and other clinical indicators. Importantly, this composite axis increases exponentially with age, predicts mortality and frailty, and remains significant even after adjusting for chronological age, yet it does not map cleanly onto specific chronic diseases (Cohen 2016). The authors interpret this pattern as evidence of an “emergent physiological process” (EPP) arising from network-level regulatory pathways, in which molecular systems gradually lose the ability to return to homeostasis due to the complex multilevel interactions. For example, another study discussed that although conserved pathways such as insulin/IGF-1 signaling and mTOR modulate lifespan in short-lived laboratory models, cross-species comparisons show that similar molecular networks operate within profoundly different outcomes (Cohen 2018). The same signaling architecture can produce steep senescence in mammals, negligible senescence in certain reptiles, or extended functional stability in colonial organisms.

Unlike a disease with a defined onset, aging instead represents a multivariate shift in organismal regulation. This also supports the hypothesis that, in humans, with extremely heterogeneous lifestyles, targeted therapeutic interventions might help delay aging but not halt or reverse it. Overall, the identification of molecular correlates of aging does not necessarily establish their causal role in organismal aging (Rattan 2020).

Moreover, Baudisch demonstrated that aging must be understood along two independent dimensions: the pace of aging, reflecting life expectancy and life-history tempo, and the shape of aging, describing how mortality changes relative to standardized lifespan (Baudisch 2011). Across species ranging from birds and mammals to primates, pace and shape do not covary in a predictable manner. For example, some long-lived birds exhibit relatively gradual increases in mortality, whereas certain short-lived mammals show steep acceleration in mortality after reaching maturity. Conversely, humans, despite being among the longest-lived mammals, show a pronounced proportional increase in late-life mortality when standardized to life expectancy (Baudisch 2011). In contrast, other taxa, such as certain reptiles, fish, and invertebrates, exhibit negligible or even negative senescence, with mortality remaining constant or declining across adult ages. Such results echo the work of Roper et al., which expanded on this concept. By recalculating age-specific selection gradients using a stable age distribution and reproductive values for 475 species, the authors demonstrated that the evolutionary force of selection does not universally decline with age (Roper et al. 2021). In many species, particularly those with stage-structured life histories or indeterminate growth, selection gradients can remain stable or even increase in later life stages – once again, reflecting the role of environmental context in shaping the aging process (Roper et al. 2021).

In humans, the emergence of long-lived populations provides a compelling demographic argument against reducing aging to a disease (Colchero et al. 2016). Analyzing extensive data across human populations and nonhuman primates, research showed that longevity follows a highly regular pattern linking pace and shape. In humans, increases in life expectancy are consistently accompanied by decreases in lifespan inequality, forming a remarkably linear relationship that spans populations with life expectancy as low as two years during crises to contemporary populations exceeding 85 years. Importantly, even under extreme mortality shocks, such as famines or epidemics, the relationship between life expectancy and lifespan equality remains intact. Mortality may temporarily surge, but the underlying coupling between pace and shape persists (Colchero et al. 2016).

Classifying aging as a disease would imply redefining species-typical trajectories as pathological states. This conclusion becomes problematic when considering species that exhibit negligible senescence or no clear functional decline with age.

A useful contrast to understand the nuances of classifying aging as a disease is obesity (Table 1). WHO classifies obesity as a disease because it represents a measurable physiological deviation that increases the risk of morbidity and mortality, even when shaped by environmental context. While it may sound similar to aging, obesity is neither species-typical (i.e., expected) nor inevitable within a population. Animals in their natural habitat will not eventually become obese.

**Table 1:** Comparison of different biological criteria between aging and other diseases

Criterion	Aging	Alzheimer’s disease	Hypertension	Type 2 diabetes	Ischemia	Cancer	Obesity
Clear subnormal biological part-function	No (near-universal, shaped by environmental contexts)	Yes (neurodegeneration beyond regular neurological decline with age)	Yes (persistent elevation of blood pressure beyond physiological range)	Yes (chronic dysregulation of glucose homeostasis)	Yes (acute interruption of tissue perfusion)	Yes (failure of cell cycle regulatory control)	Yes (chronic dysregulation of energy balance and adipose tissue accumulation)
Defined pathophysiology	Heterogeneous, context-dependent remodeling. No onset	α-Amyloid formation, tau aggregation, synaptic loss, loss of neuronal tissue density	Vascular remodeling, endothelial dysfunction	Insulin resistance, β-cell dysfunction, chronic inflammation	Hypoxia-induced metabolic failure and necrosis	Genomic instability, uncontrolled proliferation, evasion of apoptosis	Adipocyte hypertrophy/hyperplasia, insulin resistance, chronic low-grade inflammation
Diagnostic boundary	No clear pathological threshold	Established clinical and biomarker criteria	Defined blood pressure thresholds	Glycemic thresholds (HbA1c, fasting glucose)	Defined by vascular occlusion and tissue damage	Histopathological and molecular diagnostic criteria	BMI ≥ 30 kg/m^2^; additional metabolic criteria
Occurrence independent of age	Does not apply (intrinsically time-dependent)	No (although age-associated, not inevitable)	Yes	Yes	Yes (rare in children and adolescents, but possible)	Yes	Yes
Reversibility	No evidence for systemic reversal (interventions may only delay)*	Limited but targetable	Therapeutically modulated	Therapeutically modulated	Time-sensitive intervention possible	Therapeutically modulated (variable prognosis)	Yes (lifestyle, pharmacological, and surgical interventions)
Inevitable occurrence across individuals	Near-universal, though phenotypes differ	No	No	No	No	No	No
Evolutionary basis	Yes, it can be traced to primitive eukaryotes	No	No	No	No	No	No

The comparison is intended to clarify conceptual distinctions, not to capture the complexity of disease

^*^Partial reversal of specific molecular or tissue-level features, such as increased DNA repair, telomere elongation, or cosmetic interventions, for example, does not constitute reversal of organismal aging as a systemic biological process

While it is associated with increased disease risk, aging does not constitute a deviation from normative physiological and molecular strategies, nor does it present a clearly demarcated pathological onset. In this sense, Rattan proposed that organismal health is better conceptualized in terms of “homeodynamic space”, defined as the dynamic capacity of biological systems to tolerate, repair, and adapt to molecular and physiological disturbances (Rattan 2013). Within this context, aging reflects the gradual reduction of this buffering capacity, which is better aligned with evolutionary perspectives. Unlike obesity, which can occur independently of age and may be prevented or reversed without altering the organism's fundamental structure, aging unfolds as an intrinsic temporal dimension of that trajectory.

Moreover, another example of the heterogeneity of aging trajectories is that even tissues do not age at the same rate or exhibit the expected molecular response to functional stress. The brain is a particularly illustrative case.

Despite its high metabolic demand and sustained exposure to reactive oxygen species, neurons maintain tightly regulated redox control, suggesting that oxidative stress in the aging brain acts less as a primary driver and more as a threshold-dependent amplifier once compensatory capacity is exceeded (Feltes 2025a). This is further supported by a study showing that although oxidative stress can increase α-synuclein accumulation in oligodendroglial cells, it cannot drive this process (Riedel et al. 2009). Hence, even under experimentally induced cytotoxic stress, canonical molecular signatures commonly associated with neurodegeneration do not invariably emerge in a self-sustaining manner. Moreover, from an energetic standpoint, the persistence of adult hippocampal neurogenesis (Moreno-Jiménez et al. 2019), NAD⁺ recycling (Xie et al. 2020), ketone body utilization (Cunnane et al. 2020), pyruvate-mediated antioxidant buffering (Desagher et al. 1997), and compensatory hormonal strategies, well beyond the reproductive peak (Lord et al. 2008; Mosconi et al. 2024), challenges the expectation that post-reproductive maintenance should be evolutionarily minimized, as proposed by the DST (Feltes 2025b). Thus, the brain does show a hierarchically organized redistribution of metabolic resources, selectively preserving circuits essential for cognition and social adaptation in key brain regions. Importantly, neurons do not exhibit a defined replicative lifespan, as they are uncoupled from an organism's lifespan (Magrassi et al. 2013).

Similarly, DNA damage, traditionally viewed as purely deleterious (Freitas and De Magalhães 2011), has been functionally repurposed in neurons, where controlled DNA double-strand breaks mediated by TOP2B facilitate rapid transcriptional responses required for memory formation (Madabhushi et al. 2015). Despite continuous exposure to endogenous genotoxic stress and repeated cycles of activity-induced DNA breaks, neuronal populations can remain viable and functionally integrated for decades. Not only do they remain viable, but nuclear DNA fragments can also activate TLR9-mediated inflammatory pathways, further aiding memory formation (Jovasevic et al. 2024).

Another work argues that physiological liver aging is also characterized by distinct molecular alterations compared to those in other organs (Morsiani et al. 2019). Specifically, it reports increased expression of miR-31-5p, miR-141-3p, and miR-200c-3p after age 60, along with genome-wide DNA methylation remodeling that progresses up to approximately that age and then plateaus. Clinically, multiple transplantation studies indicate that livers from aged donors exhibit function and long-term survival comparable to those from younger donors, with successful use of organs from donors ≥ 80 years old and even centenarian livers (Morsiani et al. 2019). Finally, the article notes that liver aging is accelerated by chronic inflammation, which, in turn, might lead to pathological conditions. Hence, it reinforces the distinction between physiological aging and pathology arising from sustained inflammatory stress.

In the absence of a consistent pathological trajectory, the molecular alterations observed during aging are better interpreted as context-dependent regulatory shifts rather than as markers of a pathology (Rattan 2014). This argument becomes even more compelling when one recognizes that the hallmarks of aging do not correspond to the hallmarks of pathology.

## Why the hallmarks of aging and aging clocks do not justify disease classification

The identification of conserved molecular hallmarks of aging has been interpreted by some as evidence that aging is a disease.

Hallmarks are easily identifiable characteristics used to understand a given biological context. Aging-associated pathways, including genomic instability, epigenetic alterations, and altered nutrient sensing, are components of evolved life-history architecture (López-Otín et al. 2013, 2023). Their modulation may influence healthspan, but their existence does not redefine aging as pathology. Furthermore, the same molecular pathways operate across species exhibiting profoundly different senescence trajectories. If identical hallmarks can underlie negligible senescence in one taxon and steep mortality acceleration in another, then these mechanisms cannot constitute disease per se.

A good example is the hallmarks of cancer. The hallmarks of cancer were formulated to summarize the capabilities that enable malignant cells to overcome organismal control. These include sustained proliferative signaling, evasion of growth suppressors, resistance to apoptosis, replicative immortality, angiogenesis, invasion, metastasis, immune evasion, and phenotypic plasticity (Hanahan 2022). In sum, they expand cellular autonomy at the expense of tissue integrity and organismal survival and have a clear onset; hence, cancer clearly qualifies as a disease (Table 1).

From an evolutionary perspective, cancer has been described as a deviation from the normal functioning of multicellular systems (Athena Aktipis et al. 2015; Maley et al. 2017). Normal tissues enforce regulatory boundaries that limit proliferation and maintain differentiation. Neoplasia emerges when such dynamics fail. Tumorigenesis then proceeds through clonal evolution, driven by recurrent, fitness-enhancing somatic alterations and selection among competing cellular lineages (Greaves and Maley 2012; Vogelstein et al. 2013).

The hallmarks of aging describe something fundamentally different. The original basis identified genomic instability, telomere attrition, epigenetic alterations, proteostasis loss, dysregulated nutrient sensing, mitochondrial dysfunction, cellular senescence, stem cell exhaustion, and altered intercellular communication as recurrent biological features of aging (López-Otín et al. 2013). However, such hallmarks reflect the cumulative consequences of imperfect maintenance under declining selection gradients and energetic trade-offs. Even when aging increases vulnerability to disease, the trajectory itself reflects species-typical patterns.

The therapeutic logic that reinforces most arguments classifying aging as a disease further exposes the asymmetry. Clinical oncology seeks to block proliferative signaling, restore apoptosis, inhibit angiogenesis, prevent metastasis, and reactivate immune surveillance (Hanahan 2022). These interventions target a pathological process with a clear onset and progression, both of which are absent in aging. Thus, the existence of identifiable molecular traits in aging is not analogous to a disease.

Another line of argument frequently invoked in support of classifying aging as a disease draws on the literature on epigenetic clocks. DNA methylation–based estimators of biological age have emerged as among the most precise quantitative predictors. The original multi-tissue epigenetic clock developed by Horvath demonstrated that chronological age can be predicted with high accuracy across a wide range of human tissues using methylation levels at a defined set of CpG sites (Horvath 2013). Importantly, deviations between predicted epigenetic age and chronological age, often termed epigenetic age acceleration, have been associated with mortality risk, multiple age-related conditions, and functional decline. Because such parameters appear to quantify biological aging and be used to predict mortality risk, they are sometimes interpreted as evidence that aging behaves like a pathological process. As such, they capture correlational signatures of aging rather than identifying causal molecular mechanisms underlying aging. Indeed, in later works, the authors explain that the molecular mechanisms underlying these methylation changes remain incompletely understood and may reflect a combination of developmental programs, cumulative environmental exposures, and systemic physiological remodeling (Horvath and Raj 2018). Consequently, epigenetic clocks should be interpreted primarily as measures of organismal aging dynamics rather than as direct evidence of a discrete pathological process.

The predictive capacity of epigenetic clocks was further extended with the development of second-generation models such as GrimAge, which integrates methylation-based proxies for plasma proteins and smoking exposure to predict lifespan and healthspan outcomes (Lu et al. 2019). However, this predictive power should not be conflated with evidence that aging itself constitutes a disease. GrimAge and related models quantify risk and physiological remodeling associated with aging, and that individuals differ in the rate at which these changes accumulate. Yet these associations remain largely statistical, as they indicate correlation between methylation signatures and health outcomes rather than demonstrating that the underlying molecular changes constitute a pathological process. In this sense, epigenetic clocks reinforce that aging unfolds gradually over time and leaves detectable molecular traces that can be quantified.

Hence, measurement and classification are conceptually distinct problems. Just as blood pressure predicts cardiovascular events without being identical to cardiovascular disease, epigenetic age acceleration can signal elevated risk or altered physiological trajectories without implying that aging itself constitutes a pathological condition.

## Why does the debate over the medicalization of aging persist?

Although aging is fundamentally a biological and cognitive challenge, it is experienced primarily as a social phenomenon, since individuals age alongside those around them. As a result, considerations about evolutionary mechanisms or cascades of molecular events rarely occupy a central place in public discourse.

The Global Burden of Diseases, Injuries, and Risk Factors Study (GBD) from 1990–2021 found that age-associated diseases ranked among the leading causes of non-communicable disease mortality worldwide (Naghavi et al. 2024). The scientific literature is full of evidence demonstrating that aging is the primary driver of increased risk for a wide range of diseases, making an exhaustive listing of supporting studies unnecessary. Nevertheless, substantial research indicates that socioeconomic factors, particularly income, play a critical role in shaping mortality from age-related diseases (Marengoni et al. 2011). Variables such as housing stability, access to healthcare resources, and familiarity with digital platforms can significantly influence health-related decisions and outcomes (Feigin et al. 2019; Cheng et al. 2020; Jenkins Morales and Robert 2022; Lu et al. 2022; Naghavi et al. 2024; Chen et al. 2024). Hence, you cannot discuss aging without considering that economic factors determine whether an individual can afford a given treatment or even have access to adequate healthcare, both at the individual and governmental levels.

In this sense, conditions in which the risk increases greatly with age, such as dementia, heart failure, kidney failure, and osteoarthritis, account for billions of dollars in health system expenditure across numerous countries, and it is expected to continue to rise (Huang et al. 2022; Hawker and King 2022; Savarese et al. 2023; Chesnaye et al. 2024). The economic impact of caring for elderly patients within the health system is so great that it correlates negatively with a country's economic growth (Prince et al. 2015; Tang et al. 2022).

Therefore, it is not surprising that debates about how health and insurance institutions should support older adults are widespread. Aging populations use more health services and incur higher care costs, raising concerns about the sustainability of current insurance models and premium structures, especially as demographic shifts increase the proportion of older beneficiaries, often translating into higher premiums or out-of-pocket costs for older individuals and families (Kallestrup-Lamb et al. 2024). In Brazil’s private insurance market, for example, pricing rules that limit premium variation by age can lead to intergenerational transfers and strain the system as policyholders age (Santos et al. 2019).

Studies indicate that insurance coverage and financial assistance can lower out-of-pocket costs and improve access to care among older adults, emphasizing the role of policy in mitigating economic barriers to health care (Chen et al. 2023; Kallestrup-Lamb et al. 2024). Hence, promoting healthier lifestyles and effective preventive care is critical not only for individual health but also for reducing the economic burden on insurers and public health systems.

Consequently, contemporary discourse has become increasingly focused on the concept of “delayed” aging, which is more accurately understood as extending the period of life spent in good health rather than slowing the biological process of aging itself. From an economic perspective, delaying the onset of age-related diseases is projected to yield substantial financial benefits, with estimates suggesting trillions of dollars in savings by 2050 (Goldman 2016). Nonetheless, while age-associated diseases impose significant economic burdens on healthcare systems and public institutions, they may simultaneously generate economic benefits for other sectors, highlighting the sometimes-conflicting incentives embedded in aging-related health policies.

It is clear that the emergence of new diagnoses is closely linked to economic expansion in the healthcare sector, as numerous examples show that the recognition of new conditions drives the development of novel drugs, medical devices, and therapeutic interventions (MacIlwaine 2004). However, while this perspective may open new avenues for treating well-characterized diseases, it does not translate seamlessly to aging. Slowing specific biological processes associated with aging through pharmacological interventions is fundamentally different from “curing” aging itself, a notion that lacks a clear biological foundation.

Most arguments in favor of classifying aging as a disease rely on the premise that it is driven by identifiable biological factors, which we discussed before, that are not linear correlations with disease hallmarks. These arguments are further supported by the availability of robust model organisms and experimental approaches that enable systematic investigation of aging phenotypes and their modulation (Bulterijs et al. 2015). Nevertheless, despite the proliferation of startups and enterprises claiming to be on the verge of curing aging, no substantial clinical breakthroughs have been achieved to date. Instead, the most consistent and reproducible gains have come from interventions that modulate healthspan, most of which are based on lifestyle changes, which can be supported by some pharmacological interventions (Kennedy et al. 2014; The Lancet Healthy Longevity 2022).

Most compounds under investigation, including rapamycin and its analogs, senolytics, and NAD⁺ precursors, are being evaluated through repurposing strategies and are primarily tested for effects on age-associated diseases (Guarente et al. 2024). Consequently, while some interventions show context-specific health benefits, current human evidence does not demonstrate a direct halting or reversal of the biological aging process. In this sense, the growing medicalization of age-associated chronic conditions expands healthcare demand and costs without proportional gains in population health, raising serious concerns about the sustainability of public health and entitlement systems (Olshansky et al. 2005; Bloom et al. 2015). Similarly, classifying aging as a disease does not imply that insurance will cover medical expenditures based on “old age” simply because it was labeled a pathology (Boorse 2025).

Hence, lifestyle interventions remain the most effective, safe, and translationally validated strategies for improving healthspan in humans (Longo et al. 2015; Rattan 2020). Framing aging as a disease risks expanding medicalization and healthcare costs without proportional gains in population health, particularly in already strained public systems. A more pragmatic and evidence-based approach is to address age-associated diseases, reduce socioeconomic barriers to care, prioritize policies that promote healthy aging, and narrow the debate between longevity science and clinicians rather than pursuing the ill-defined goal of curing aging itself (The Lancet Healthy Longevity 2022).

Finally, while the growing interest in classifying aging as a disease is partly driven by the notion that doing so could facilitate access to dedicated funding mechanisms and clinical trial pathways traditionally structured around clearly defined pathologies, the proposal may also reflect increased institutional incentives to accelerate translational research. Nevertheless, these classification issues should remain distinct from biological criteria, because they lack a scientific basis. The absence of formal disease classification does not diminish the importance of studying aging as a biologically consequential process. Age-associated functional decline remains the primary risk factor for most chronic conditions and represents a major determinant of morbidity and mortality worldwide. Understanding its mechanisms and identifying strategies to preserve physiological resilience, therefore, remain central objectives of biomedical research, irrespective of whether aging is formally defined as a disease.

## Conclusion: scientific caution in the midst of translational enthusiasm

Aristotle framed aging as a natural process marked by the gradual loss of an internal “vital heat” or life force (Aristotle 1984). Although modern biology has long moved beyond this simplified explanation, Aristotle’s formulation captured an essential intuition of his time. Despite centuries of scientific progress, most people's everyday understanding of aging remains, as expected, grounded in lived experience rather than in molecular or evolutionary explanations. While this notion has little impact on longevity science itself, it strongly shapes how aging is portrayed to the public and to institutions, influencing expectations and fueling often enthusiastic and overly optimistic promises about what will “soon” be available to treat aging-related conditions. Moreover, oversimplifying aging profoundly influences how flawed theories spread rapidly among the public, fostering unrealistic expectations about which interventions can genuinely extend lifespan.

A good example is the often mistaken view that aging is a rate-limited process governed by a single physiological feature, such as the long-discredited notion of a fixed number of heartbeats per lifetime. In fact, rate-of-living theories have long been discredited and lack an evolutionary basis, yet they somehow found their way into the present (Speakman et al. 2002; Speakman 2005). Such reductionist views impose artificial limits on a multifactorial process and misguide expectations.

Unfortunately, this recurring pattern is not confined to ancient worldviews. Modern biomedical history provides multiple examples of aging theories that gained rapid visibility before being substantially revised. The free radical theory of aging, once presented as a near-universal explanatory theory (Harman 1956, 2003), gradually lost its status as a unifying mechanism due to no increased life expectancy in model organisms after modulating antioxidant defenses (Liochev 2013) and when antioxidant supplementation failed to reproducibly extend lifespan in humans and often yielded neutral or even adverse outcomes in clinical trials (Miller et al. 2005; Ristow et al. 2009). It is clear that aging is intrinsically associated with increased reactive oxygen species generation (Barja 2014; Viña 2019), but aging is not driven by redox imbalance, which reflects a recurring tendency to elevate individual molecular pathways to disproportionately central roles in aging biology.

Across evolutionary data, aging emerges not as a pathology but as a species-typical, time-dependent remodeling process centered within an environmental/social context. Although aging increases vulnerability to disease and shares certain molecular features with pathological states, these overlaps do not collapse the conceptual boundary between biological progression and pathological deviation. Aging unfolds as a near-universal trajectory, lacking a discrete onset, a singular mechanistic architecture, and a clear therapeutic target.

Recent ethical analyses of longevity science have compellingly argued that efforts to mitigate age-related decline should be grounded in respect for autonomy, self-ownership, and the intrinsic value of life, rather than solely in economic projections (Han and de Magalhães 2026). Such reasoning rightly places the protection of human flourishing at the center of the debate. At the same time, however, the ethical legitimacy of pursuing healthspan-extending interventions does not eliminate the need for conceptual precision. Whether aging fulfills the biological criteria required for disease classification remains a distinct debate.

Likewise, a recent study identified several unresolved questions spanning evolutionary theory, organismal survival dynamics, and the heterogeneity of aging phenotypes (Rattan 2024). These include the unclear identity and universality of longevity-assurance pathways, the mechanisms governing biological time across species, and the determinants of variability in aging trajectories from the molecular to the organismal level. Importantly, age-associated changes cannot always be interpreted as harmful processes, as many alterations may reflect adaptive remodeling, compensatory responses, or hormetic stress responses that contribute to organismal resilience. Hence, the scientific community must recognize that the architecture of aging remains incompletely characterized, and caution against premature attempts to frame aging within rigid pathological classifications.

Recognizing this distinction does not diminish the importance of geroscience or of pursuing interventions to extend healthspan. On the contrary, theoretical clarity strengthens translational efforts by preserving the distinction between intrinsic biological aging and age-related diseases. Under current evolutionary and biological evidence, classifying aging itself as a disease lacks scientific basis. Even well-intentioned narratives can divert scientific effort and inflate public expectations.

## Data Availability

No datasets were generated or analysed during the current study.

## References

[CR1] The World Health Organization (2025) Ageing and health. https://www.who.int/news-room/fact-sheets/detail/ageing-and-health

[CR2] Amorim JA, Coppotelli G, Rolo AP et al (2022) Mitochondrial and metabolic dysfunction in ageing and age-related diseases. Nat Rev Endocrinol 18:243–258. 10.1038/s41574-021-00626-7 35145250 10.1038/s41574-021-00626-7 PMC9059418

[CR3] Araya-Ajoy YG, Westneat DF, Wright J (2020) Pathways to social evolution and their evolutionary feedbacks. Evolution (n Y) 74:1894–1907. 10.1111/evo.14054 10.1111/evo.14054 32627189

[CR4] Aristotle (1984) On Youth and Old Age, On Life and Death, On Breathing. In: Barnes J (ed) The Complete Works of Aristotle, Vol. 1. Princeton: Princeton University Press, p 1264

[CR5] Athena Aktipis C, Boddy AM, Jansen G et al (2015) Cancer across the tree of life: Cooperation and cheating in multicellularity. Philos Trans R Soc B Biol Sci. 10.1098/rstb.2014.0219 10.1098/rstb.2014.0219 PMC4581024 26056363

[CR6] Barja G (2014) The Mitochondrial Free Radical Theory of Aging. In: Osiewacz HD (ed) Progress in Molecular Biology and Translational Science. Elsevier, pp 1–27 10.1016/B978-0-12-394625-6.00001-5 25149212

[CR7] Baudisch A (2011) The pace and shape of ageing. Methods Ecol Evol 2:375–382. 10.1111/j.2041-210X.2010.00087.x

[CR8] Bellary S, Kyrou I, Brown JE, Bailey CJ (2021) Type 2 diabetes mellitus in older adults: clinical considerations and management. Nat Rev Endocrinol 17:534–548. 10.1038/s41574-021-00512-2 34172940 10.1038/s41574-021-00512-2

[CR9] Bloom DE, Chatterji S, Kowal P et al (2015) Macroeconomic implications of population ageing and selected policy responses. Lancet 385:649–657. 10.1016/S0140-6736(14)61464-1 25468167 10.1016/S0140-6736(14)61464-1 PMC4469267

[CR10] Boorse C (1977) Health as a theoretical concept. Philos Sci 44:542–573. 10.1086/288768

[CR11] Boorse C (2014) A second rebuttal on health. J Med Philos (United Kingdom) 39:683–724. 10.1093/jmp/jhu035 10.1093/jmp/jhu035 25398760

[CR12] Boorse C (2025) Boundaries of disease: vagueness and overdiagnosis. J Med Philos (United Kingdom) 50:231–247. 10.1093/jmp/jhaf001 10.1093/jmp/jhaf001 40285504

[CR13] Bulterijs S, Hull RS, Björk VCE, Roy AG (2015) It is time to classify biological aging as a disease. Front Genet 6:1–5. 10.3389/fgene.2015.00205 26150825 10.3389/fgene.2015.00205 PMC4471741

[CR14] Chen J, Zhao M, Zhou R et al (2023) How heavy is the medical expense burden among the older adults and what are the contributing factors? A literature review and problem-based analysis. Front Public Health 11:1–8. 10.3389/fpubh.2023.1165381 10.3389/fpubh.2023.1165381 PMC10313336 37397714

[CR15] Chen S, Cao Z, Nandi A et al (2024) The global macroeconomic burden of Alzheimer’s disease and other dementias: estimates and projections for 152 countries or territories. Lancet Glob Health 12:e1534–e1543. 10.1016/S2214-109X(24)00264-X 39151988 10.1016/S2214-109X(24)00264-X

[CR16] Cheng X, Yang Y, Schwebel DC et al (2020) Population ageing and mortality during 1990-2017: a global decomposition analysis. PLoS Med 17:1–17. 10.1371/journal.pmed.1003138 10.1371/journal.pmed.1003138 PMC7279585 32511229

[CR17] Chesnaye NC, Ortiz A, Zoccali C et al (2024) The impact of population ageing on the burden of chronic kidney disease. Nat Rev Nephrol 20:569–585. 10.1038/s41581-024-00863-9 39025992 10.1038/s41581-024-00863-9

[CR18] Cohen AA (2016) Complex systems dynamics in aging: new evidence, continuing questions. Biogerontology 17:205–220. 10.1007/s10522-015-9584-x 25991473 10.1007/s10522-015-9584-x PMC4723638

[CR19] Cohen AA (2018) Aging across the tree of life: the importance of a comparative perspective for the use of animal models in aging. Biochim Biophys Acta Mol Basis Dis 1864:2680–2689. 10.1016/j.bbadis.2017.05.028 28690188 10.1016/j.bbadis.2017.05.028

[CR20] Colchero F, Rau R, Jones OR et al (2016) The emergence of longevous populations. Proc Natl Acad Sci U S A 113:E7681–E7690. 10.1073/pnas.1612191113 27872299 10.1073/pnas.1612191113 PMC5137748

[CR21] Cunnane SC, Trushina E, Morland C et al (2020) Brain energy rescue: an emerging therapeutic concept for neurodegenerative disorders of ageing. Nat Rev Drug Discov 19:609–633. 10.1038/s41573-020-0072-x 32709961 10.1038/s41573-020-0072-x PMC7948516

[CR22] da Costa JP, Vitorino R, Silva GM et al (2016) A synopsis on aging—theories, mechanisms and future prospects. Ageing Res Rev 29:90–112. 10.1016/j.arr.2016.06.005 27353257 10.1016/j.arr.2016.06.005 PMC5991498

[CR23] Desagher S, Glowinski J, Prémont J (1997) Pyruvate protects neurons against Hydrogen Peroxide-induced toxicity. J Neurosci 17:9060–9067. 10.1523/JNEUROSCI.17-23-09060.1997 9364052 10.1523/JNEUROSCI.17-23-09060.1997 PMC6573585

[CR24] Epel ES, Prather AA (2018) Stress, telomeres, and psychopathology: toward a deeper understanding of a triad of early aging. Annu Rev Clin Psychol 14:371–397. 10.1146/annurev-clinpsy-032816-045054 29494257 10.1146/annurev-clinpsy-032816-045054 PMC7039047

[CR25] Feigin VL, Nichols E, Alam T et al (2019) Global, regional, and national burden of neurological disorders, 1990–2016: a systematic analysis for the global burden of disease study 2016. Lancet Neurol 18:459–480. 10.1016/S1474-4422(18)30499-X 30879893 10.1016/S1474-4422(18)30499-X PMC6459001

[CR26] Feltes BC (2025a) Analyzing different aging theories in the context of the brain: DNA damage, inflammation, redox imbalance, and neurodevelopment intertwine. Biogerontology 26:105. 10.1007/s10522-025-10243-w 40323483 10.1007/s10522-025-10243-w

[CR27] Feltes BC (2025b) Selective resilience in brain aging: challenging the scope of the disposable soma theory. Biogerontology 26:1–13. 10.1007/s10522-025-10292-1 10.1007/s10522-025-10292-1 40748396

[CR28] Freitas AA, De Magalhães JP (2011) A review and appraisal of the DNA damage theory of ageing. Mutat Res Rev Mutat Res 728:12–22. 10.1016/j.mrrev.2011.05.001 10.1016/j.mrrev.2011.05.001 21600302

[CR29] Gems D (2015) The aging-disease false dichotomy: understanding senescence as pathology. Front Genet 6:1–7. 10.3389/fgene.2015.00212 26136770 10.3389/fgene.2015.00212 PMC4468941

[CR30] Goldman D (2016) The economic promise of delayed aging. Cold Spring Harb Perspect Med. 10.1101/cshperspect.a025072 10.1101/cshperspect.a025072 PMC4743067 26684333

[CR31] Greaves M, Maley CC (2012) Clonal evolution in cancer. Nature 481:306–313. 10.1038/nature10762 22258609 10.1038/nature10762 PMC3367003

[CR32] Guarente L, Sinclair DA, Kroemer G (2024) Human trials exploring anti-aging medicines. Cell Metab 36:354–376. 10.1016/j.cmet.2023.12.007 38181790 10.1016/j.cmet.2023.12.007

[CR33] Han ZZ, de Magalhães JP (2026) The ethics case for longevity science. Ageing Res Rev. 10.1016/j.arr.2026.103054 41654143 10.1016/j.arr.2026.103054

[CR34] Hanahan D (2022) Hallmarks of cancer: new dimensions. Cancer Discov 12:31–46. 10.1158/2159-8290.CD-21-1059 35022204 10.1158/2159-8290.CD-21-1059

[CR35] Harman D (1956) Aging: a theory based on free radical and radiation chemistry. J Gerontol 11:298–300. 10.1093/geronj/11.3.298 13332224 10.1093/geronj/11.3.298

[CR36] Harman D (2003) The free radical theory of aging. Antioxid Redox Signal 5:557–561. 10.1089/152308603770310202 14580310 10.1089/152308603770310202

[CR37] Hawker GA, King LK (2022) The burden of osteoarthritis in older adults. Clin Geriatr Med 38:181–192. 10.1016/j.cger.2021.11.005 35410675 10.1016/j.cger.2021.11.005

[CR38] The World Health Organization (2020) Healthy ageing and functional ability. https://www.who.int/news-room/questions-and-answers/item/healthy-ageing-and-functional-ability

[CR39] Horvath S, Raj K (2018) DNA methylation-based biomarkers and the epigenetic clock theory of ageing. Nat Rev Genet. 10.1038/s41576-018-0004-3 29643443 10.1038/s41576-018-0004-3

[CR40] Horvath S (2013) DNA methylation age of human tissues and cell types DNA methylation age of human tissues and cell types

[CR41] Huang Y, Li X, Liu Z et al (2022) Projections of the economic burden of care for individuals with dementia in mainland China from 2010 to 2050. PLoS ONE 17:1–13. 10.1371/journal.pone.0263077 10.1371/journal.pone.0263077 PMC8812891 35113895

[CR42] Jenkins Morales M, Robert SA (2022) Housing cost burden and health decline among low- and moderate-income older renters. J Gerontol B Psychol Sci Soc Sci 77:815–826. 10.1093/geronb/gbab184 34622283 10.1093/geronb/gbab184 PMC8974342

[CR43] Jones OR, Scheuerlein A, Salguero-Gómez R et al (2014) Diversity of ageing across the tree of life. Nature 505:169–173. 10.1038/nature12789 24317695 10.1038/nature12789 PMC4157354

[CR44] Jovasevic V, Wood EM, Cicvaric A et al (2024) Formation of memory assemblies through the DNA-sensing TLR9 pathway. Nature 628:145–153. 10.1038/s41586-024-07220-7 38538785 10.1038/s41586-024-07220-7 PMC10990941

[CR45] Kallestrup-Lamb M, Marin AOK, Menon S, Søgaard J (2024) Aging populations and expenditures on health. J Econ Ageing. 10.1016/j.jeoa.2024.100518

[CR46] Kennedy BK, Berger SL, Brunet A et al (2014) Geroscience: linking aging to chronic disease. Cell 159:709–713. 10.1016/j.cell.2014.10.039 25417146 10.1016/j.cell.2014.10.039 PMC4852871

[CR47] Kirkwood TBL (1977) Evolution of ageing. Nature 270:301–304. 10.1038/270301a0 593350 10.1038/270301a0

[CR48] Kivimäki M, Pentti J, Frank P et al (2025) Social disadvantage accelerates aging. Nat Med 31:1635–1643. 10.1038/s41591-025-03563-4 40087516 10.1038/s41591-025-03563-4 PMC12092251

[CR49] Lemoine M (2020) Defining aging. Biol Philos 35:1–30. 10.1007/s10539-020-09765-z

[CR50] Li S, Vazquez JM, Sudmant PH (2023) The evolution of aging and lifespan. Trends Genet 39:830–843. 10.1016/j.tig.2023.08.005 37714733 10.1016/j.tig.2023.08.005 PMC11147682

[CR51] Liochev SI (2013) Reactive oxygen species and the free radical theory of aging. Free Radic Biol Med 60:1–4. 10.1016/j.freeradbiomed.2013.02.011 23434764 10.1016/j.freeradbiomed.2013.02.011

[CR52] Liu Z, Liang Q, Ren Y et al (2023) Immunosenescence: molecular mechanisms and diseases. Signal Transduct Target Ther 8:200. 10.1038/s41392-023-01451-2 37179335 10.1038/s41392-023-01451-2 PMC10182360

[CR53] Longevity TLH (2022) Is ageing a disease? The Lancet. Healthy Longevity 3(7):e448. 10.1016/S2666-7568(22)00154-4 36102754 10.1016/S2666-7568(22)00154-4

[CR54] Longo VD, Antebi A, Bartke A et al (2015) Interventions to slow aging in humans: are we ready? Aging Cell 14:497–510. 10.1111/acel.12338 25902704 10.1111/acel.12338 PMC4531065

[CR55] López-Otín C, Blasco MA, Partridge L et al (2013) The hallmarks of aging. Cell 153:1194. 10.1016/j.cell.2013.05.039 23746838 10.1016/j.cell.2013.05.039 PMC3836174

[CR56] López-Otín C, Blasco MA, Partridge L et al (2023) Hallmarks of aging: an expanding universe. Cell 186:243–278. 10.1016/j.cell.2022.11.001 36599349 10.1016/j.cell.2022.11.001

[CR57] Lord C, Buss C, Lupien SJ, Pruessner JC (2008) Hippocampal volumes are larger in postmenopausal women using estrogen therapy compared to past users, never users and men: a possible window of opportunity effect. Neurobiol Aging 29:95–101. 10.1016/j.neurobiolaging.2006.09.001 17030472 10.1016/j.neurobiolaging.2006.09.001

[CR58] Lu AT, Quach A, Wilson JG, Reiner AP, Aviv A, Raj K, Hou L, Baccarelli AA, Li Y, Stewart JD, Whitsel EA (2019) DNA methylation GrimAge strongly predicts lifespan and healthspan. Aging (albany NY) 11(2):303 30669119 10.18632/aging.101684 PMC6366976

[CR59] Lu X, Yao Y, Jin Y (2022) Digital exclusion and functional dependence in older people: findings from five longitudinal cohort studies. eClinicalMedicine 54:101708. 10.1016/j.eclinm.2022.101708 36353265 10.1016/j.eclinm.2022.101708 PMC9637559

[CR60] MacIlwaine SW (2004) What is a disease? EMBO Rep 5:650–653. 10.1136/bmj.2.2085.170315229637

[CR61] Madabhushi R, Gao F, Pfenning AR et al (2015) Activity-induced DNA breaks govern the expression of neuronal early-response genes. Cell 161:1592–1605. 10.1016/j.cell.2015.05.032 26052046 10.1016/j.cell.2015.05.032 PMC4886855

[CR62] Magrassi L, Leto K, Rossi F (2013) Lifespan of neurons is uncoupled from organismal lifespan. Proc Natl Acad Sci U S A 110:4374–4379. 10.1073/pnas.1217505110 23440189 10.1073/pnas.1217505110 PMC3600460

[CR63] Maley CC, Aktipis A, Graham TA et al (2017) Classifying the evolutionary and ecological features of neoplasms. Nat Rev Cancer 17:605–619. 10.1038/nrc.2017.69 28912577 10.1038/nrc.2017.69 PMC5811185

[CR64] Marengoni A, Angleman S, Melis R et al (2011) Aging with multimorbidity: a systematic review of the literature. Ageing Res Rev 10:430–439. 10.1016/j.arr.2011.03.003 21402176 10.1016/j.arr.2011.03.003

[CR65] Medawar P (1953) Unsolved problem of biology. Med J Aust 1:854–855. 10.5694/j.1326-5377.1953.tb84985.x 13062953 10.5694/j.1326-5377.1953.tb84985.x

[CR66] Menassa M, Stronks K, Khatmi F et al (2023) Concepts and definitions of healthy ageing: a systematic review and synthesis of theoretical models. eClinicalMedicine 56:101821. 10.1016/j.eclinm.2022.101821 36684393 10.1016/j.eclinm.2022.101821 PMC9852292

[CR67] Miller ER, Pastor-Barriuso R, Dalal D et al (2005) Meta-analysis: high-dosage vitamin E supplementation may increase all-cause mortality. Ann Intern Med 142:37–46. 10.7326/0003-4819-142-1-200501040-00110 15537682 10.7326/0003-4819-142-1-200501040-00110

[CR68] Moreno-Jiménez EP, Flor-García M, Terreros-Roncal J et al (2019) Adult hippocampal neurogenesis is abundant in neurologically healthy subjects and drops sharply in patients with Alzheimer’s disease. Nat Med 25:554–560. 10.1038/s41591-019-0375-9 30911133 10.1038/s41591-019-0375-9

[CR69] Morsiani C, Bacalini MG, Santoro A et al (2019) The peculiar aging of human liver: a geroscience perspective within transplant context. Ageing Res Rev 51:24–34. 10.1016/j.arr.2019.02.002 30772626 10.1016/j.arr.2019.02.002

[CR70] Mosconi L, Nerattini M, Matthews DC et al (2024) In vivo brain estrogen receptor density by neuroendocrine aging and relationships with cognition and symptomatology. Sci Rep 14:1–15. 10.1038/s41598-024-62820-7 38902275 10.1038/s41598-024-62820-7 PMC11190148

[CR71] Naghavi M, Ong KL, Aali A et al (2024) Global burden of 288 causes of death and life expectancy decomposition in 204 countries and territories and 811 subnational locations, 1990–2021: a systematic analysis for the global burden of disease study 2021. Lancet 403:2100–2132. 10.1016/S0140-6736(24)00367-2 38582094 10.1016/S0140-6736(24)00367-2 PMC11126520

[CR72] Nichols E, Steinmetz JD, Vollset SE et al (2022) Estimation of the global prevalence of dementia in 2019 and forecasted prevalence in 2050: an analysis for the global burden of disease study 2019. Lancet Public Health 7:e105–e125. 10.1016/S2468-2667(21)00249-8 34998485 10.1016/S2468-2667(21)00249-8 PMC8810394

[CR73] The World Health Organization (2026) Old age. https://www.who.int/standards/classifications/frequently-asked-questions/old-age

[CR74] Olshansky SJ, Passaro DJ, Hershow RC et al (2005) A potential decline in life expectancy in the United States in the 21st century. N Engl J Med 352:1138–1145. 10.1056/nejmsr043743 15784668 10.1056/NEJMsr043743

[CR75] Pamplona R, Jové M, Gómez J, Barja G (2023) Programmed versus non-programmed evolution of aging. what is the evidence? Exp Gerontol. 10.1016/j.exger.2023.112162 37004927 10.1016/j.exger.2023.112162

[CR76] Prince MJ, Wu F, Guo Y et al (2015) The burden of disease in older people and implications for health policy and practice. Lancet 385:549–562. 10.1016/S0140-6736(14)61347-7 25468153 10.1016/S0140-6736(14)61347-7

[CR77] Rattan SIS (2013) Healthy ageing, but what is health? Biogerontology 14:673–677. 10.1007/s10522-013-9442-7 23852043 10.1007/s10522-013-9442-7

[CR78] Rattan SIS (2014) Aging is not a disease: implications for intervention. Aging Dis 5:196–202. 10.14336/AD.2014.0500196 24900942 10.14336/AD.2014.0500196 PMC4037311

[CR79] Rattan SIS (2020) Naive extrapolations, overhyped claims and empty promises in ageing research and interventions need avoidance. Biogerontology 21:415–421. 10.1007/s10522-019-09851-0 31773357 10.1007/s10522-019-09851-0

[CR80] Rattan SIS (2024) Seven knowledge gaps in modern biogerontology. Biogerontology 25:1–8. 10.1007/s10522-023-10089-0 38206540 10.1007/s10522-023-10089-0

[CR81] Riedel M, Goldbaum O, Richter-Landsberg C (2009) α-Synuclein promotes the recruitment of tau to protein inclusions in oligodendroglial cells: effects of oxidative and proteolytic stress. J Mol Neurosci 39:226–234 19266322 10.1007/s12031-009-9190-y

[CR82] Ristow M, Zarse K, Oberbach A et al (2009) Antioxidants prevent health-promoting effects of physical exercise in humans. Proc Natl Acad Sci U S A 106:8665–8670. 10.1073/pnas.0903485106 19433800 10.1073/pnas.0903485106 PMC2680430

[CR83] Roper M, Capdevila P, Salguero-Gómez R (2021) Senescence: Why and where selection gradients might not decline with age. Proc R Soc B Biol Sci. 10.1098/rspb.2021.0851 10.1098/rspb.2021.0851 PMC8292751 34284628

[CR84] Rose MR, Flatt T, Graves JL et al (2012) What is aging? Front Genet 3:2011–2013. 10.3389/fgene.2012.00134 10.3389/fgene.2012.00134 PMC3400891 22833755

[CR85] Santos SL, Turra CM, Noronha K (2019) Population aging and health spending: an analysis of intergenerational and intragenerational transfers in the Brazilian private health care plans. Rev Bras Estud Popul 35:1–30. 10.20947/S102-3098A0062

[CR86] Savarese G, Becher PM, Lund LH et al (2023) Global burden of heart failure: a comprehensive and updated review of epidemiology. Cardiovasc Res 118:3272–3287. 10.1093/cvr/cvac013 35150240 10.1093/cvr/cvac013

[CR87] Speakman JR (2005) Body size, energy metabolism and lifespan. J Exp Biol 208:1717–1730. 10.1242/jeb.01556 15855403 10.1242/jeb.01556

[CR88] Speakman JR, Selman C, McLaren JS, Harper EJ (2002) Living fast, dying when? The link between aging and energetics. J Nutr 132:1583S-1597S. 10.1093/jn/132.6.1583S 12042467 10.1093/jn/132.6.1583S

[CR89] Tang B, Li Z, Hu S, Xiong J (2022) Economic implications of health care burden for elderly population. Inq (United States). 10.1177/00469580221121511 10.1177/00469580221121511 PMC9445535 36062304

[CR90] The World Health Organization (2019) International Classification of Diseases for Mortality and Morbidity Statistics (11th Revision) – ICD-11. Geneva

[CR91] Trastus LA, d’Adda di Fagagna F (2025) The complex interplay between aging and cancer. Nat Aging 5:350–365. 10.1038/s43587-025-00827-z 40038418 10.1038/s43587-025-00827-z PMC7618899

[CR92] Vaupel JW (2010) Biodemography of human ageing. Nature 464:536–542. 10.1038/nature08984 20336136 10.1038/nature08984 PMC4010874

[CR93] Viña J (2019) The free radical theory of frailty: mechanisms and opportunities for interventions to promote successful aging. Free Radic Biol Med 134:690–694. 10.1016/j.freeradbiomed.2019.01.045 30735838 10.1016/j.freeradbiomed.2019.01.045

[CR94] Vogelstein B, Papadopoulos N, Velculescu VE et al (2013) Cancer genome landscapes. Science 339:1546–1558. 10.1126/science.1235122 23539594 10.1126/science.1235122 PMC3749880

[CR95] Williams GC (1957) Pleiotropy, natural selection, and the evolution of senescence. Sci Aging Knowl Environ 11:398–411. 10.1126/sageke.2001.1.cp13

[CR96] World Health Organization (2015) World Report on Ageing and Health. Geneva

[CR97] Xie N, Zhang L, Gao W et al (2020) NAD+ metabolism: pathophysiologic mechanisms and therapeutic potential. Signal Transduct Target Ther. 10.1038/s41392-020-00311-7 33028824 10.1038/s41392-020-00311-7 PMC7539288

[CR98] Zhavoronkov A, Bhullar B (2015) Classifying aging as a disease in the context of ICD-11. Front Genet 6:1–8. 10.3389/fgene.2015.00326 26583032 10.3389/fgene.2015.00326 PMC4631811

